# Abnormal gut microbiota composition contributes to cognitive dysfunction in SAMP8 mice

**DOI:** 10.18632/aging.101464

**Published:** 2018-06-10

**Authors:** Gaofeng Zhan, Ning Yang, Shan Li, Niannian Huang, Xi Fang, Jie Zhang, Bin Zhu, Ling Yang, Chun Yang, Ailin Luo

**Affiliations:** 1 Department of Anesthesiology, Tongji Hospital, Tongji Medical College, Huazhong University of Science and Technology, Wuhan, China; 2 Department of Cardiology and Critical Care Medicine, The Third Affiliated Hospital of Soochow University, Suzhou, China; * Equal contribution

**Keywords:** cognitive dysfunction, gut microbiota, Alzheimer’s disease, SAMP8, pseudo germ-free mice

## Abstract

Alzheimer’s disease is characterized by cognitive dysfunction and aging is an important predisposing factor; however, the pathological and therapeutic mechanisms are not fully understood. Recently, the role of gut microbiota in Alzheimer’s disease has received increasing attention. The cognitive function in senescence-accelerated mouse prone 8 (SAMP8) mice was significantly decreased and the Chao 1 and Shannon indices, principal coordinates analysis, and principal component analysis results were notably abnormal compared with that of those in senescence-accelerated mouse resistant 1 (SAMR1) mice. Moreover, 27 gut bacteria at six phylogenetic levels differed between SAMP8 and SAMR1 mice. In a separate study, we transplanted fecal bacteria from SAMP8 or SAMR1 mice into pseudo germ-free mice. Interestingly, the pseudo germ-free mice had significantly lower cognitive function prior to transplant. Pseudo germ-free mice that received fecal bacteria transplants from SAMR1 mice but not from SAMP8 mice showed improvements in behavior and in α-diversity and β-diversity indices. In total, 14 bacteria at six phylogenetic levels were significantly altered by the gut microbiota transplant. These results suggest that cognitive dysfunction in SAMP8 mice is associated with abnormal composition of the gut microbiota. Thus, improving abnormal gut microbiota may provide an alternative treatment for cognitive dysfunction and Alzheimer’s disease.

## Introduction

Alzheimer’s disease (AD) is a neurodegenerative disease that occurs in the elderly [1,2]. Its clinical manifestation includes progressive cognitive dysfunction and memory impairment [1,3]. AD leads to the death of a large number of neural cells, damaging memory, cognitive ability, and ultimately basic self-care and physiological function, until death [4]. In addition to aging symptoms, senescence-accelerated mouse prone 8 (SAMP8) mice show aging-related learning and memory impairment and cognitive dysfunction and are thus commonly used as model animals in AD studies [5,6].

Gut microbiota refers to the large number of microorganisms that coexist in the digestive tract and are an integral part of the body [7]. The role of the gut microbiota is not limited to the gastrointestinal tract, and gut microbiota can have a major impact on brain function and behavior through the three routes of the gut–brain axis (immune, neuroendocrine, and vagal pathways) [8,9]. Furthermore, the gut microbiota have been implicated in many diseases [7,10]. Studies on germ-free mice have demonstrated that the abnormal composition of the gut microbiota can cause behavior deficits and suppress brain function and neural biochemical characteristics [11,12]. We previously reported that gut *Bifidobacteria* might confer resilience to chronic social defeat stress [13] and that oral intake of *Bifidobacteria* facilitated alleviation of depression symptoms [13]. The differential antidepressant effects of *R*-ketamine and *S*-ketamine may be attributed to the differential profiles of the gut microbiota [14]. These findings provide a causal link between the gut and brain where the gut microbiota are an important remote mediator of brain function that affects the development and therapeutic outcomes of brain diseases.

Recently, several studies have suggested significant interactions between alterations of the gut microbiota and cognitive behavior [15,16]. Harach et al. [17] used 16S rRNA gene sequencing to analyze and compare differences in the composition of the gut microbiota between amyloid precursor protein (APP), presenilin 1 (PS1), and age-matched healthy wild-type (WT) mice. They found that APP and PS1 mice had significantly less bacteria, proteobacteria, and actinomycetes but more *Bacteroidetes* and *Trichomonas* compared with the WT group. In a study by Jiang et al., germ-free mice showed significantly increased brain Aβ levels after transplant of fecal samples from AD mice; however, fecal transplants from WT mice had no effect on behavioral or biomedical outcomes [18]. Histological changes in the brain and alterations in the gut microbiota composition have been observed in AD mice at different ages [19]. Notable differences have also been reported in the gut microbiota between patients with AD and healthy individuals [20], suggesting that abnormal composition of the gut microbiota results in the appearance of AD symptoms.

The present study was conducted to compare the composition of the gut microbiota between SAMP8 and senescence-accelerated mouse resistant 1 (SAMR1) mice. Furthermore, we examined the effects of SAMP8 and SAMR1 mice fecal bacteria transplants on the cognitive behavior of antibiotic-induced pseudo germ-free mice.

## RESULTS

### Comparison of cognitive behavior between SAMR1 and SAMP8 mice

**Figure 1:**
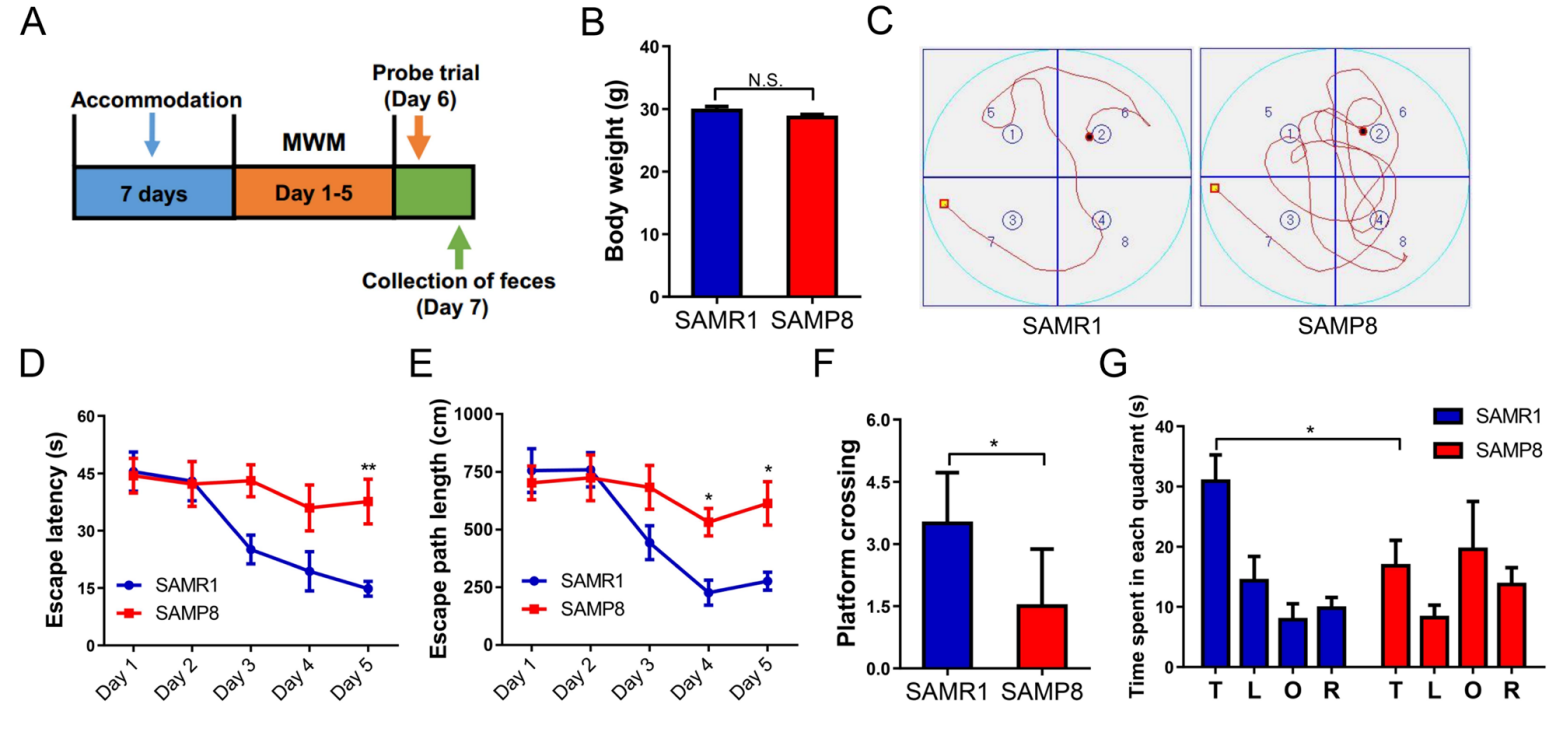
**MWMT for SAMR1 and SAMP8 mice.** (**A**) The schedule for MWMT and fecal sample collection. Seven days after accommodation, SAMR1 and SAMP8 mice were scheduled for MWMT and probe trial on days 1–6. On day 7, fecal samples were collected for 16S rRNA gene sequencing and fecal bacteria transplant. (**B**) Body weight (T-test, *P* > 0.05). (**C**) The trace graph of SAMR1 and SAMP8 mice in MWMT. (**D**) Escape latency (two-way ANOVA; Time: F_4,20_ = 6.062, *P* < 0.01; Group: F_1,5_ = 8.802, *P* < 0.05; Interaction: F_4,20_ = 3.476, *P* < 0.05). (**E**) Escape path length (two-way ANOVA. Time: F_4,20_ = 11.32, *P* < 0.001; Group: F_1,5_ = 4.834, *P* = 0.0792; Interaction: F_4,20_ = 3.271, *P* < 0.05). (**F)** Platform crossing (*t*-test, *P* < 0.05). (**G**) Time spent in each quadrant (*t*-test, *P* < 0.05). ANOVA: analysis of variance; MWMT: Morris water maze test. Data are shown as mean ± SEM (n = 6). *P < 0.05, **P < 0.01.

It is well recognized that diet, weight, and environment have a great impact on the gut microbiota [21]. We first determined that there was no significant difference in body weight between SAMR1 and SAMP8 mice (Fig. 1B). Next, we evaluated cognitive behavior in the two groups using the Morris water maze test (MWMT) (Fig. 1C-G). SAMP8 mice demonstrated a significantly increased escape latency and path length on day 5 (Fig. 1D, E). In the probe trial, platform crossing and time spent in each quadrant were both significantly lower for SAMP8 mice than for SAMR1 mice (Fig. 1F, G).

### α-diversity and β-diversity of the gut microbiota in SAMR1 and SAMP8 mice

**Figure 2:**
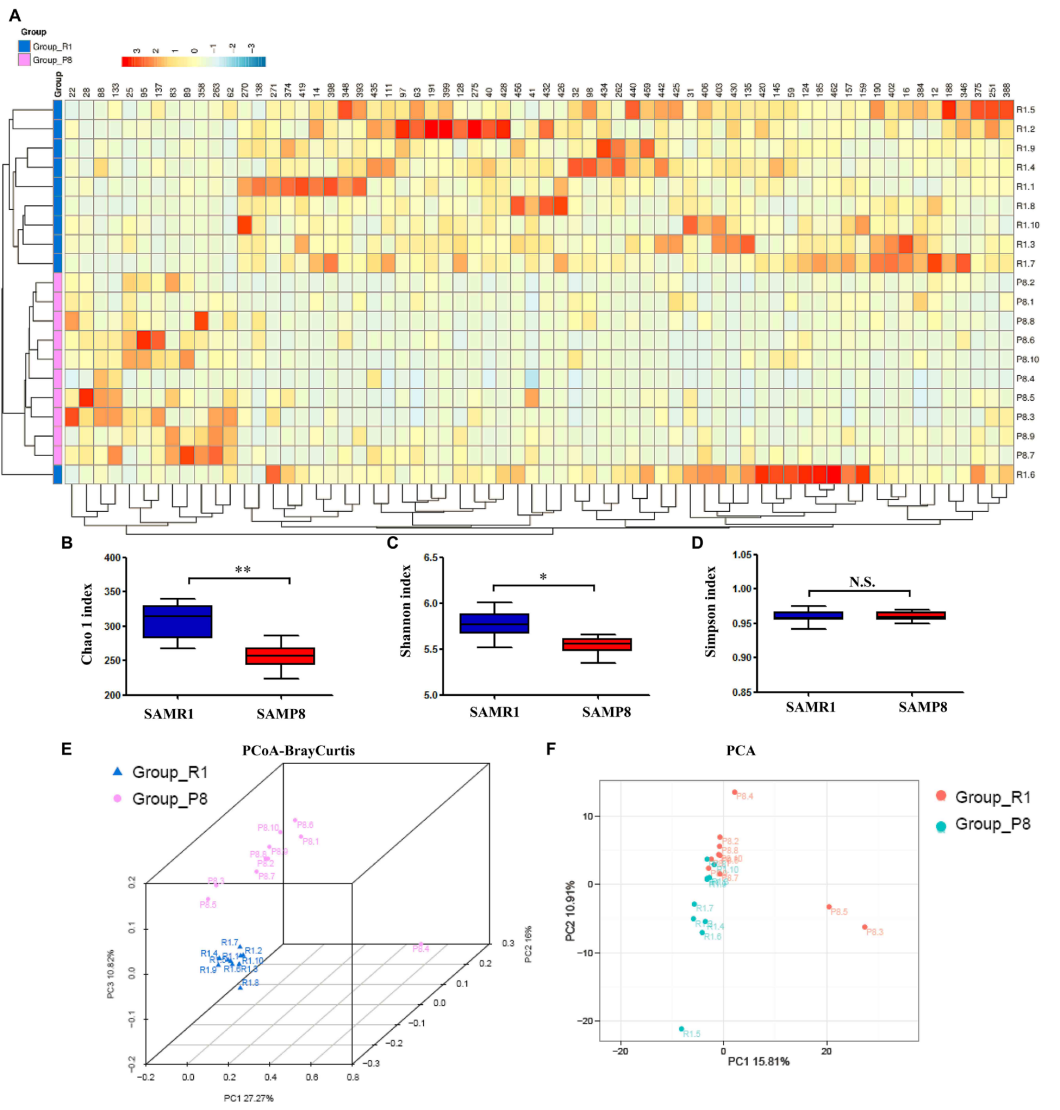
**Differential profiles of the gut microbiota between SAMR1 and SAMP8 mice.** (**A**) Heat map of differential levels of bacteria between the groups. (**B**) Chao 1 index (*t*-test, *P* < 0.01). (**C**) Shannon index (*t*-test, *P* < 0.05). (**D**) Simpson index (*t*-test, *P* > 0.05). (**E**) PCoA analysis of gut bacteria data (Bray–Curtis dissimilarity). (**F**) PCA analysis of gut bacteria data. α-diversity data are shown as mean ± SEM (n = 10). PCA: principal component analysis; PCoA: principal coordinates analysis. *P < 0.05, **P < 0.01.

α-diversity refers to the diversity of bacteria or species within a community or habitat and is mainly concerned with the number of bacteria or species therein [22]. The Chao 1, Shannon, and Simpson indices are commonly used to evaluate the α-diversity of microbiota [22]. The Chao 1 and Shannon indices were significantly lower in fecal samples from SAMP8 mice than from SAMR1 mice, although there was no significant difference in the Simpson index between the two groups (Fig. 2B-D). Regarding β-diversity, principal coordinates analysis (PCoA) plots of Bray–Curtis dissimilarity and primary component analysis (PCA) between the two groups showed that the dots of SAMP8 mice (P8.1–10) were not close to the dots of SAMR1 mice (R1.1–10) (Fig. 2E, F). Thus, it is likely that SAMP8 mice have a different gut microbiota composition than SAMR1 mice.

### Alterations in the gut microbiota composition between SAMR1 and SAMP8 mice

**Figure 3:**
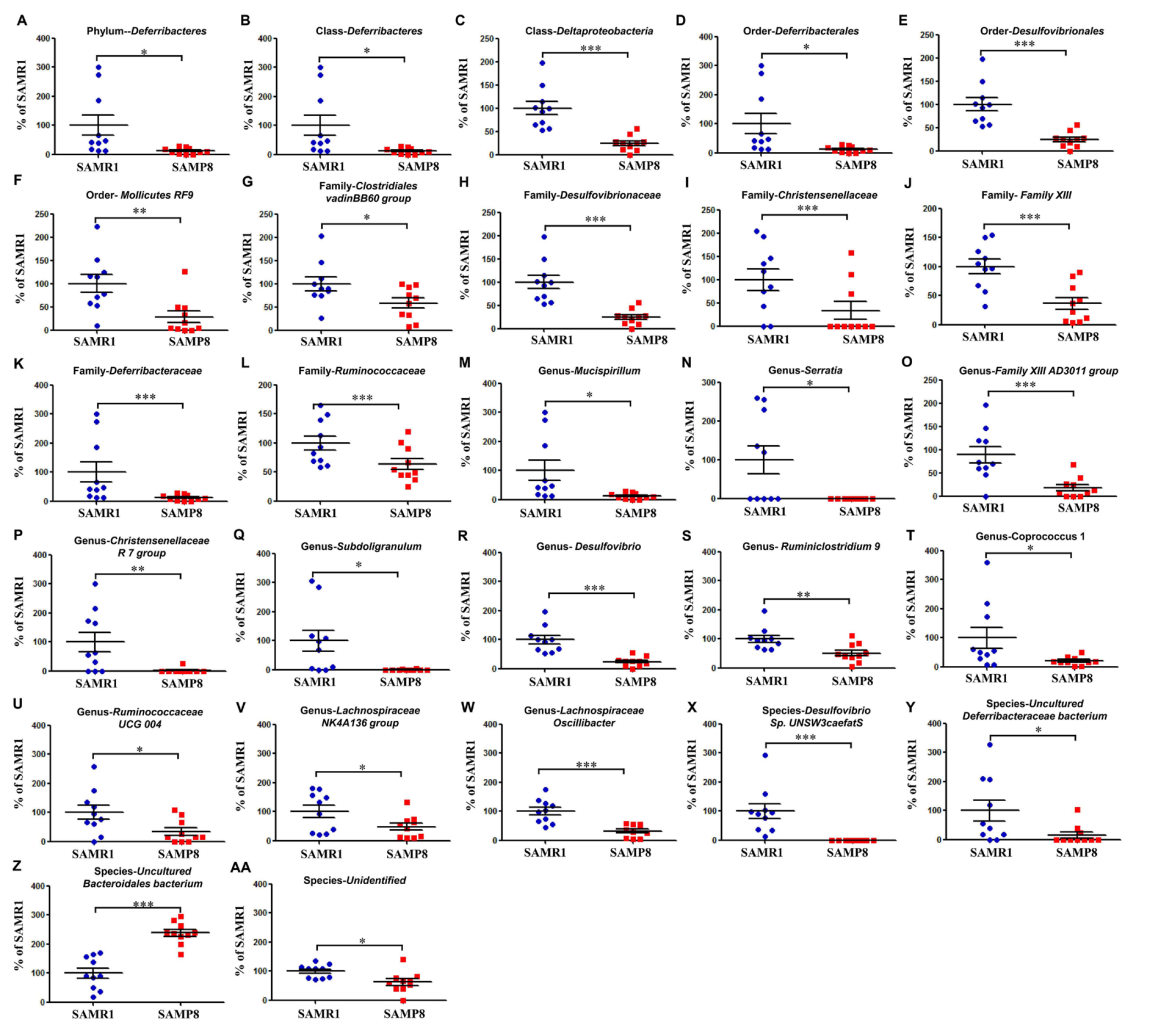
**Differential levels of the gut bacteria between SAMR1 and SAMP8 mice.** (**A**) Phylum *Deferribacteres* (*t*-test, *P* < 0.05). (**B**) Class *Deferribacteres* (*t*-test, *P* < 0.05). (**C**) Class *Deltaproteobacteria* (*t*-test, *P* < 0.001). (**D**) Order *Deferribacterales* (*t*-test, *P* < 0.05). (**E**) Order *Desulfovibrionales* (*t*-test, *P* < 0.001). (**F**) Order *Mollicutes RF9* (*t*-test, *P* < 0.01). (**G**) Family *Clostridiales vadinBB60 group* (*t*-test, *P* < 0.05). (**H**) Family *Desulfovibrionaceae* (*t*-test, *P* < 0.001). (**I)** Family *Christensenellaceae* (*t*-test, *P* < 0.001). (**J**) Family *Family XIII* (*t*-test, *P* < 0.001). (**K**) Family *Deferribacteraceae* (*t*-test, *P* < 0.001). (**L**) Family *Ruminococcaceae* (*t*-test, *P* < 0.001). (**M**) Genus *Mucispirillum* (*t*-test, *P* < 0.05). (**N**) Genus *Serratia* (*t*-test, *P* < 0.05). (**O**) Genus *Family XIII AD3011 group* (*t*-test, *P* < 0.001). (**P**) Genus *Christensenellaceae R-7 group* (*t*-test, *P* < 0.01). (**Q**) Genus *Subdoligranulum* (*t*-test, *P* < 0.05). (**R**) Genus *Desulfovibrio* (*t*-test, *P* < 0.001). (**S**) Genus *Ruminiclostridium 9* (*t*-test, *P* < 0.01). (**T**) Genus *Coprococcus 1* (*t*-test, *P* < 0.05). (**U**) Genus *Ruminococcaceae UCG 004* (*t*-test, *P* < 0.05). (**V**) Genus *Lachnospiraceae NK4A136 group* (*t*-test, *P* < 0.05). **(W**) Genus *Lachnospiraceae oscillibacter* (*t*-test, *P* < 0.001). (**X**) Species *Desulfovibrio sp. UNSW3caefatS* (*t*-test, *P* < 0.001). (**Y**) Species *uncultured Deferribacteraceae bacterium* (*t*-test, *P* < 0.05). (**Z**) Species *uncultured Bacteroidales bacterium* (*t*-test, *P* < 0.001). (**AA**) Species Unidentified (*t*-test, *P* < 0.05).

We used 16S rRNA gene sequencing to determine the alterations in the gut microbiota composition between SAMR1 and SAMP8 mice. The analysis showed that 27 bacteria differed between the fecal samples from SAMR1 and SAMP8 mice (Fig. 3A-AA). The relative abundance of 26 bacteria at six phylogenetic levels (phylum, class, order, etc.) was significantly decreased in SAMP8 mice compared with SAMR1 mice (Fig. 3A-Y, AA). In contrast, the relative abundance of the species *uncultured Bacteroidales bacterium* was significantly increased in SAMP8 mice compared with SAMR1 mice (Fig. 3Z).

### Effects of SAMR1 and SAMP8 gut microbiota transplant on MWMT behavior in antibiotic-induced pseudo germ-free mice

**Figure 4:**
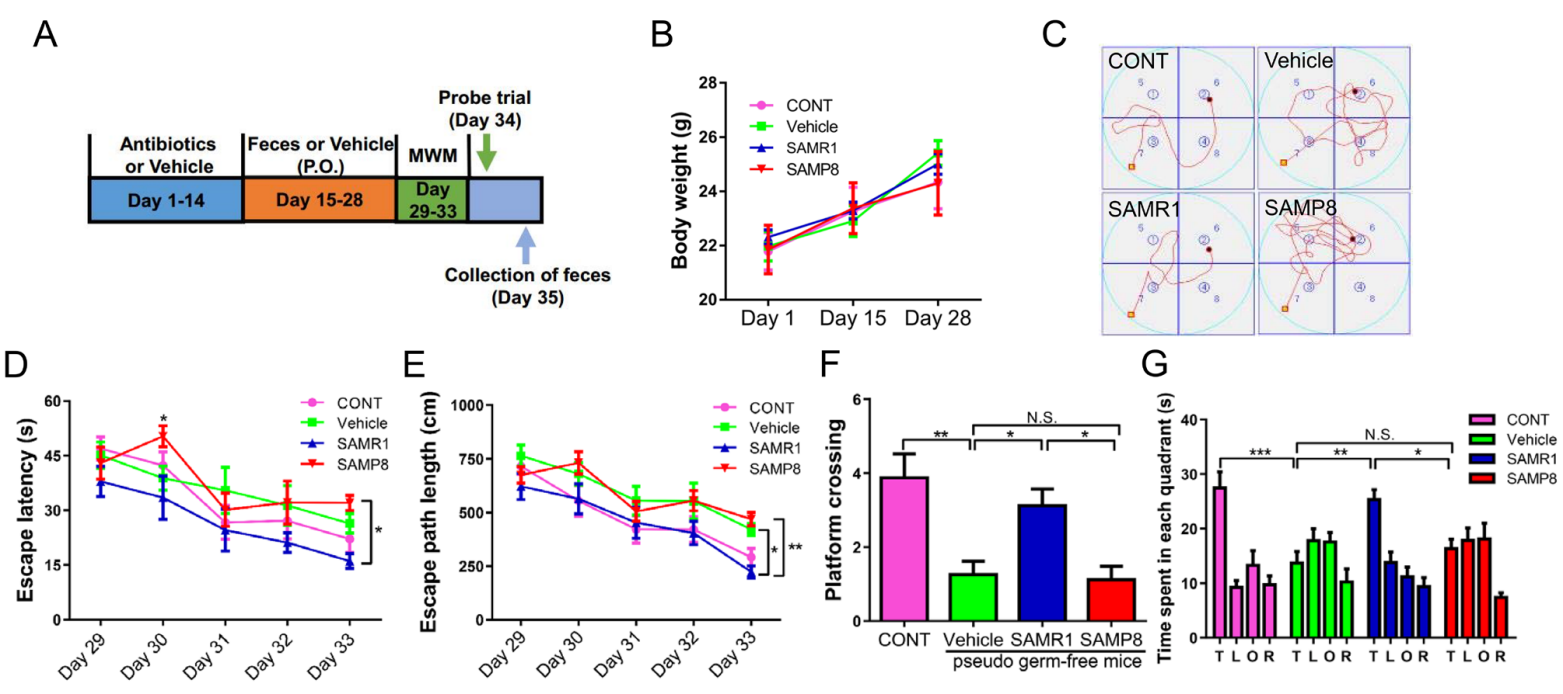
**Effects of SAMR1 and SAMP8 mice fecal microbiota transplant on behavior in pseudo germ-free mice.** (**A**) Schedule of fecal microbiota transplant on behavioral testing in pseudo germ-free mice. Mice were first treated by administering large doses of antibiotic solution for 14 consecutive days. Thereafter, mice were orally treated with fetal microbiota of SAMR1 and SAMP8 mice. MWMT was performed on days 29–33, and the probe trial was performed on day 34. On day 35, fecal samples were collected for 16S rRNA gene sequencing testing. (**B**) Body weight (Two-way ANOVA; Time: F_2,14_ = 25.59, *P* < 0.001; Group: F_3,21_ = 0.1132, *P* = 0.9514; Interaction: F_6,42_ = 0.6019, *P* = 0.7272.). (**C**) The trace graph of mice in MWMT. (**D**) Escape latency (two-way ANOVA; Time: F_4,28_ = 17.23, *P* < 0.001; Group: F_3,21_ = 3.27, *P* < 0.05; Interaction: F_12,84_ = 0.9568, *P* = 0.4961). (**E**) Escape path length (two-way ANOVA; Time: F_4,28_ = 32.04, *P* < 0.001; Group: F_3,21_ = 8.668, *P* < 0.001; Interaction: F_12,84_ = 0.7349, *P* = 0.7137.). (**F**) Platform crossing (one-way ANOVA; F_3,28_ = 8.745, *P* < 0.001.). (**G**) Time spent in each quadrant (one-way ANOVA; F_3,28_ = 9.133, *P* < 0.001). ANOVA: analysis of variance; MWMT: Morris water maze test. Data are shown as mean ± SEM (n = 8). *P < 0.05, **P < 0.01, ***P < 0.001.

The body weights of mice in the four groups showed no significant changes on days 1, 15, and 28 (Fig. 4B). A pseudo germ-free mouse model was created by administering antibiotics at large doses for 14 consecutive days. Interestingly, we found a slight increase in escape latency and path length in the vehicle-treated group compared with the control group, although there was no significant change between the two groups (Fig. 4D, E). Gut microbiota from SAMR1 and SAMP8 mice were transplanted into the gastrointestinal tract of pseudo germ-free mice for 14 consecutive days. Gut microbiota transplant from SAMR1 mice, but not from SAMP8 mice, improved abnormal behavior in MWMT. In the probe trial, gut microbiota transplant from SAMR1 mice, but not from SAMP8 mice, improved abnormalities in platform crossing results and time spent in target quadrant (Fig. 4F, G).

### Effects of SAMR1 and SAMP8 gut microbiota transplant on α-diversity and β-diversity in antibiotic-induced pseudo germ-free mice

**Figure 5:**
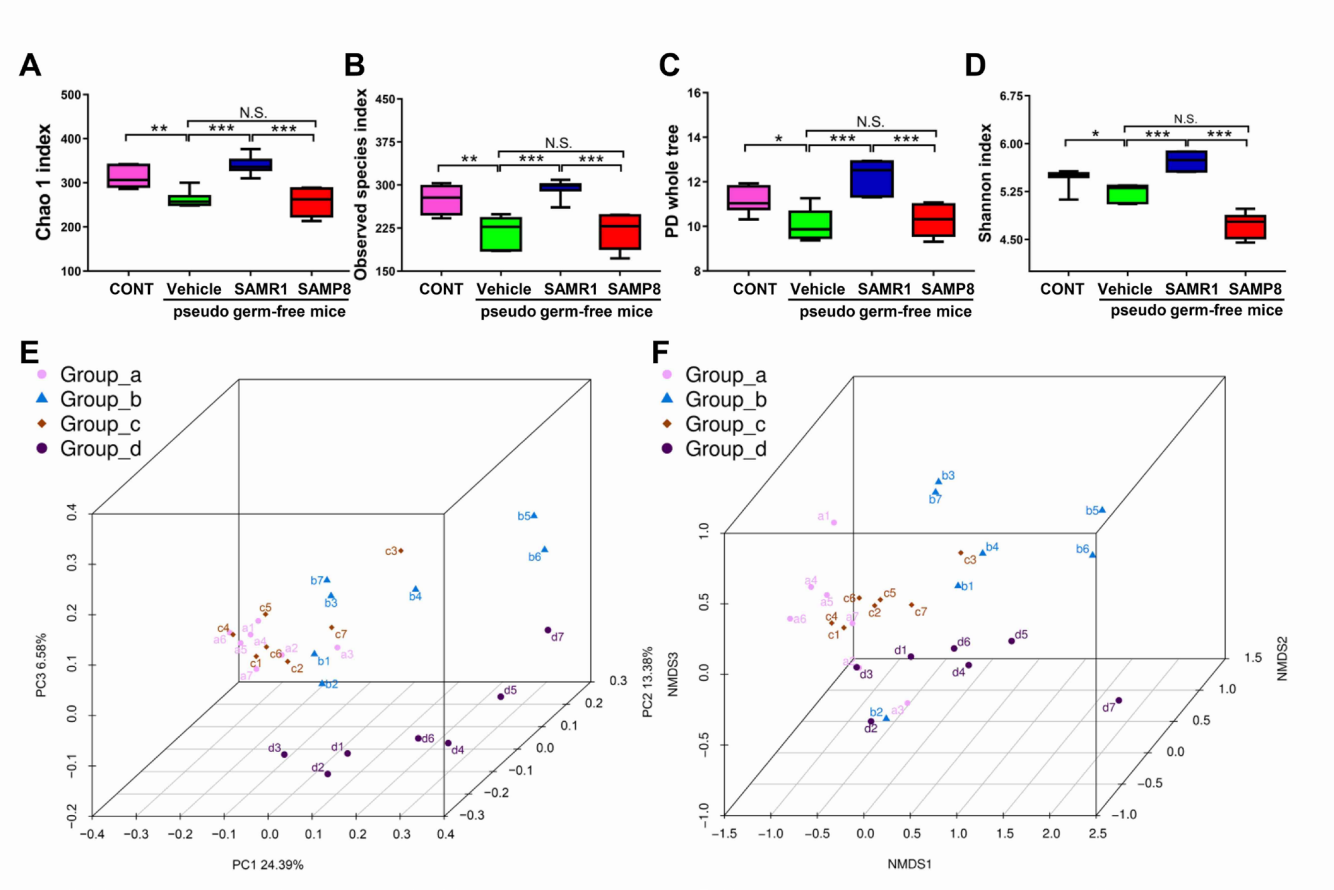
**α-diversity and β-diversity of fecal microbiota transplant in pseudo germ-free mice.** (**A**) Chao 1 index (One-way ANOVA; F_3,24_ = 21.28, *P* < 0.001). (**B**) Observed species index (One-way ANOVA; F_3,24_ = 17.29, *P* < 0.001). (**C**) PD whole tree (One-way ANOVA; F_3,24_ = 16.76, *P* < 0.001). (**D**) Shannon index (One-way ANOVA; F_3,24_ = 50.19, *P* < 0.001). Data are shown as mean ± SEM (n = 7). *P < 0.05, **P < 0.01, ***P < 0.001. (**E**) PCoA analysis of gut bacteria data (Bray–Curtis dissimilarity). (**F**) NMDS analysis of gut bacteria data. NMDS: non-metric multi-dimensional scaling; PCoA: principal coordinates analysis.

In addition to the Chao 1 and Shannon indices, we measured observed species index and PD whole tree index to fully understand the gut microbiota composition (Fig. 5A-D). Vehicle-treated pseudo germ-free mice had significantly decreased α-diversity indices, including the Chao 1, observed species index, PD whole tree, and Shannon indices, compared with the control group (Fig. 5A-D). Gut microbiota transplant from SAMR1 mice, but not from SAMP8 mice, significantly improved abnormality in α-diversity (Fig. 5A-D).

PCoA analysis plots for the four groups showed that the dots of vehicle-treated mice (b1–b7) were not close to the dots of control mice and that the dots of SAMR1 mice (c1–c7) were close to the dots of control mice (a1–a7) but not SAMP8 mice (d1–d10) (Fig. 5E, F). Thus, it is likely that the effects of fecal microbiota transplant from SAMP8 and SAMR1 mice to the host gut microbiota were significantly different.

### Effects of fecal microbiota transplant from SAMP8 and SAMR1 mice on the abundance of host gut microbiota

**Figure 6:**
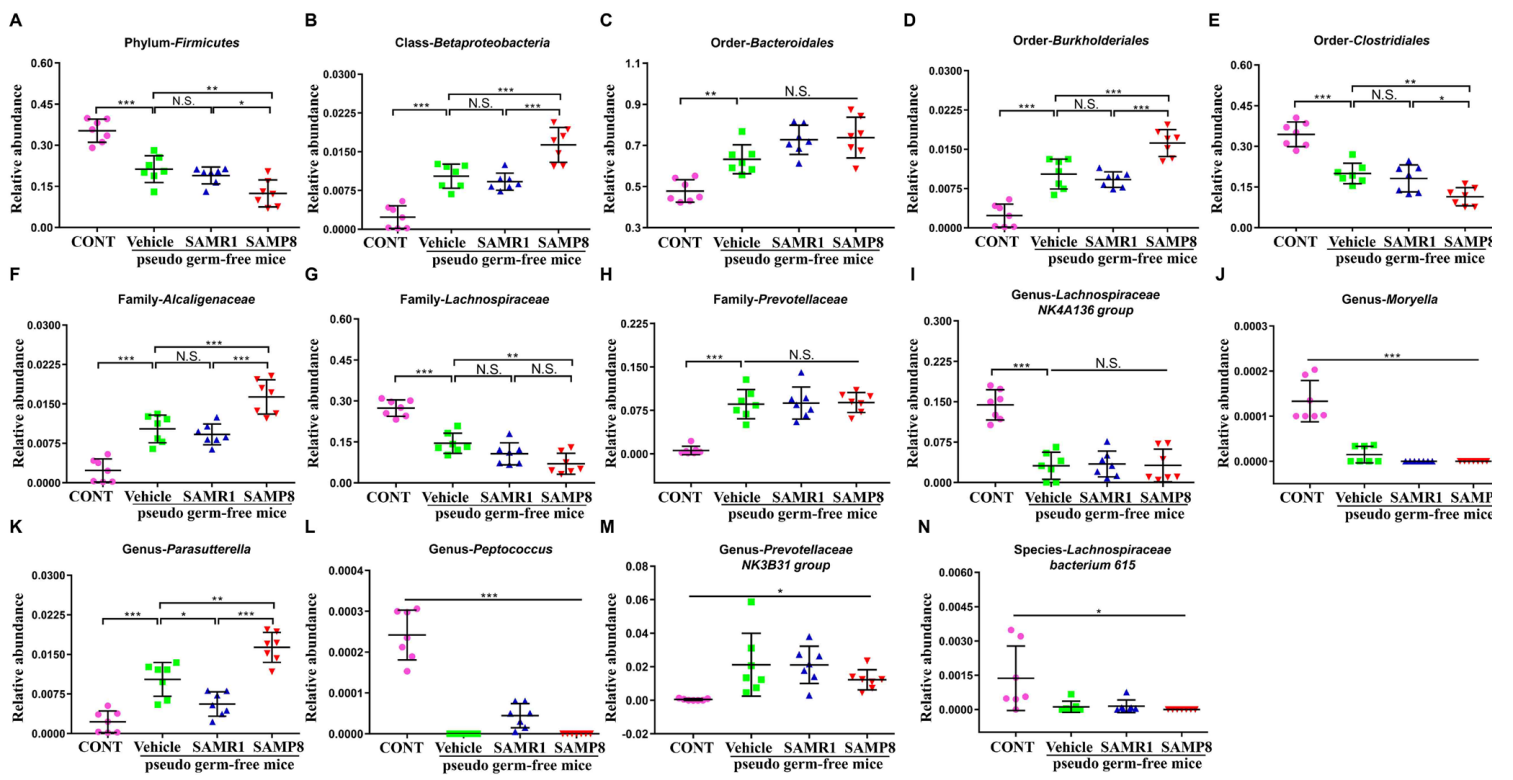
**Effects of fecal microbiota transplant on levels of gut microbiota in pseudo germ-free mice.** (**A**) Phylum *Firmicutes* (One-way ANOVA; F_3,24_ = 34.61, *P* < 0.001). (**B**) Class *Betaproteobacteria* (One-way ANOVA; F_3,24_ = 37.98, *P* < 0.001). (**C**) Order *Bacteroidales* (One-way ANOVA; F_3,24_ = 17.77, *P* < 0.001). (**D**) Order *Burkholderiales* (One-way ANOVA; F_3,24_ = 41.43, *P* < 0.001). (**E**) Order *Clostridiales* (One-way ANOVA; F_3,24_ = 36.92, *P* < 0.001). (**F**) Family *Alcaligenaceae* (One-way ANOVA; F_3,24_ = 34.95, *P* < 0.001). (**G**) Family *Lachnospiraceae* (One-way ANOVA; F_3,24_ = 40.75, *P* < 0.001). (**H**) Family *Prevotellaceae* (One-way ANOVA; F_3,24_ = 26.69, *P* < 0.001). (**I**) Genus *Lachnospiraceae NK4A136 group* (One-way ANOVA; F_3,24_ = 30.02, *P* < 0.001). (**J**) Genus *Moryella* (Fisher’s exact test; *P* < 0.001). (**K**) Genus *Parasutterella* (One-way ANOVA; F_3,24_ = 37.64, *P* < 0.001). (**L**) Genus *Peptococcus* (Fisher’s exact test; *P* < 0.001). (**M**) Genus *Prevotellaceae NK3B31 group* (Fisher’s exact test; *P* < 0.05). (**N**) Species *Lachnospiraceae bacterium 615* (Fisher’s exact test; *P* < 0.05). ANOVA: analysis of variance

A total of 14 bacteria at six levels (phylum, class, order, etc.) were significantly altered among the four groups (Fig. 6A-N). Vehicle-treated mice showed a significant change in the levels of 14 bacteria. However, SAMP8 or SAMR1 fecal microbiota transplants failed to elicit any changes in the levels of the order *Bacteroidales*, family *Prevotellaceae*, genus *Lachnospiraceae NK4A136 group*, *Moryella*, *Prevotellaceae NK3B31 group*, and species *Lachnospiraceae bacterium 615* in pseudo germ-free mice (Fig. 6C, H-J, M, N). Interestingly, SAMP8 or SAMR1 fecal microbiota transplants significantly improved or further aggravated changes in another eight bacteria in pseudo germ-free mice (Fig. 6A, B, D-G, K, L).

## DISCUSSION

MWMT is a behavioral test in which rodents learn to find a platform hidden in the water [23]. It is mainly used to measure learning and memory of spatial orientation. In the present study, we found that SAMP8 mice have deficits in spatial learning and memory, consistent with those described in previous studies [24,25]. Additionally, SAMP8 mice possessed a different gut microbiota composition compared with SAMR1 mice and fecal microbiota transplant from SAMP8 mice further aggravated performance on MWMT in antibiotic-induced pseudo germ-free mice. The results of 16S rRNA gene sequencing suggested that SAMP8 and SAMR1 fecal microbiota transplants had different effects on the composition and levels of the gut microbiota in pseudo germ-free mice. These findings suggest that abnormal gut microbiota composition is at least partially associated with the dysfunctional cognitive, learning, and memory performances in SAMP8 mice. Interventional regulation of the gut microbiota in mice may improve central nervous system (CNS) disorders or symptoms in SAMP8 mice.

Increasing evidence demonstrates that the gut microbiota are important in maintaining normal function of CNS through metabolic, neuroendocrine, and immune pathways [26]. A previous study from our group suggested that abnormality in the gut microbiota confers susceptibility to stress in a mouse model of chronic social defeat stress [13]. On the contrary, in the same animal model, the differential antidepressant actions of ketamine enantiomers could be attributed to different gut microbiota composition, which is likely caused by distinct pharmacological profiles of ketamine enantiomer [14]. Recently, there has been a focus on the causal link between gut microbiota composition and cognitive processes such as learning and memory [27]. In this study, behavioral differences between SAMP8 and SAMR1 mice were found to be related to the different composition and relative abundance of the gut microbiota, as demonstrated by significant differences in both α-diversity and β-diversity. A total of 27 bacteria were found to be significantly different between SAMP8 and SAMR1 mice. The order *Desulfovibrionales*, families *Christensenellaceae* and *Ruminococcaceae*, and genus *Desulfovibrio* are reportedly related to cognitive dysfunction in SAMP8 mice [28], which is consistent with the results of this study. Other changes in the gut microbiota might be associated with aging-related cognitive dysfunction, although this has not yet been confirmed. Collectively, the gut microbiota is indeed associated with aging-related cognitive dysfunction.

Germ-free animals are a useful experimental model for observing the effects of specific microbiota on host physiologic, metabolic, and behavioral actions [29]. Germ-free mice are widely used to study fecal microbiota transplant [30]. In this study, we used large doses of antibiotics to construct pseudo germ-free mice, rather than absolute germ-free mice, because absolute germ-free mice were not likely to carry out MWMT for 6 days in non-germ-free environments. More than 90% of the gut microbiota would be killed by antibiotics, and the behavioral performances of pseudo germ-free mice are similar to those of absolute germ-free mice [31]. Interestingly, we found that antibiotics significantly aggravated learning and memory in MWMT and that fecal microbiota transplant from SAMR1 mice, but not SAMP8 mice, exerted favorable effects on impaired cognitive function in pseudo germ-free mice, suggesting that there is a causal link between the effects of gut microbiota and cognitive function.

To the best of our knowledge, this is the first study showing the effects of SAMP8 and SAMR1 fecal microbiota transplant on spatial learning and memory. Furthermore, 16S rRNA gene sequencing implicated 14 bacteria at six levels in the effects of fecal microbiota transplant on cognitive function. It is commonly recognized that a dysfunctional level of the phylum *Firmicutes* is associated with cognitive dysfunction [32,33]. Antibiotics significantly decreased the level of the phylum *Firmicutes* and SAMP8 mice fecal microbiota transplant further decreased its level, although SAMR1 mice fecal microbiota transplant failed to affect this change. The order *Bacteroidales* is also reported to be associated with cognitive dysfunction in SAMP8 mice [28], which is consistent with the results of the present study. Additionally, *Parasutterella,* a relatively novel genus of Gram-negative bacteria, has been exclusively reported in the gut microbiota literature [34]. In this study, SAMR1 fecal microbiota transplant significantly increased levels of *Parasutterella*, while SAMP8 fecal microbiota transplant further increased it. These findings suggest that fecal microbiota transplant has an exact physiological effect on cognitive function: alterations in levels of bacteria may improve or deteriorate cognitive function.

Overall, our study suggests that abnormalities in the gut microbiota are involved in the pathogenesis of age-related cognitive impairment in SAMP8 mice. Furthermore, improving abnormal gut microbiota may provide a therapeutic target for age-related cognitive dysfunction. Subsequent large-scale studies are needed to further elucidate the role and possible mechanisms of the gut microbiota in age-related and CNS diseases.

## MATERIALS AND METHODS

### Animals

Seven-month-old male senescence-accelerated mouse resistant 1 (SAMR1, 28–31 g) and senescence-accelerated mouse prone 8 (SAMP8, 28–31 g) mice were purchased from the Biotechnology Co. Beijing Zhongkezesheng. Two-month-old male C57BL/6 mice (20–23 g) were obtained from the Animal Center of Tongji Hospital. Animals were adapted to their environmental conditions for 7 days before experiments. Animals were housed in polypropylene cages with food and water ad libitum. The room temperature was maintained at 22°C ± 2°C and a relative humidity of 60% ± 5% on a 12-h light/dark cycle. All experimental protocols and animal handling procedures were carried out in strict accordance with the recommendations in the Guide for the Care and Use of Laboratory Animals, published by the National Institutes of Health (NIH Publications No. 80-23, revised in 1996). This study was approved by the Ethical Committee on Animal Experimentation of the Tongji Hospital, Tongji Medical College, Huazhong University of Science and Technology.

### MWMT

Spatial learning and memory function were assessed using MWMT according to a previous study [35]. Mice were subjected to four trials per day for 5 consecutive days in a circular pool (120 cm diameter and 50 cm height) containing a 10-cm-diameter hidden platform, which was placed in the target quadrant and submerged 1 cm below the water surface for all trials. Each mouse was placed in the water facing the pool wall and given 60 s to locate the hidden platform. If the mice failed to find the platform within 60 s, they were gently guided to the platform and allowed to remain there for 15 s. The time and distance taken to reach the platform (escape latency) was measured, which reflects spatial learning. On the sixth day, a 60-s probe trial was performed to assess the memory of the mice. The number of times the mice crossed the platform area and the percentage of time spent in the target quadrant were recorded by a digital video camera.

### Pseudo germ-free mice modeling

Based on a previous study with slight modification [36], broad-spectrum antibiotics (ampicillin 1 g/L, neomycin sulfate 1 g/L, metronidazole 1 g/L, Sigma-Aldrich Co. Ltd, USA) dissolved in drinking water were given ad libitum to C57BL/6 mice for 14 consecutive days. The drinking solution was renewed every 2 days.

### Fecal microbiota transplant

The mice were placed in a clean cage with sterilized filter paper. The feces samples were collected immediately after defecation in a sterilized centrifuge tube. The filter paper was replaced for different mouse samples. Feces were stored in a −80°C freezer until analysis and transplant [13]. Fecal microbiota was prepared by diluting 1 g of fecal sample obtained from SAMR1 or SAMP8 mice in 10 mL of sterile PBS. The fecal material was suspended and 0.2 mL of the suspension was guided by gavage into each mouse recipient for 14 days [36].

### 16S rRNA analysis of fecal samples

The fecal samples were collected immediately after behavioral tests (Fig. 1A and Fig. 4A). Samples were placed in 1.5 ml tubes, snap-frozen on dry ice, and stored at −80°C. The 16S rRNA analysis of the fecal samples was performed by OE Biotech Co., Ltd. (Shanghai, China). DNA extraction was performed using TIANamp stool DNA kits (Tiangen Biotechnology Company, Beijing, China). Genomic DNA was amplified in 50 μL triplicate reactions with bacterial 16S rRNA gene (V3–V5 region)-specific primers: 338F (5′-ACTCCTACGGGAGGCAGC-3′) and 806R (5′-GG ACTACHVGGGTWTCTAAT-3′). The reverse primer contained a sample barcode and both primers were connected with an Illumina sequencing adapter. PCR products were purified and the concentrations were adjusted for sequencing on an Illumina Miseq PE300 system. Original sequencing reads from the sample were sorted by unique barcodes, followed by the removal of the barcode, linker, and PCR primer sequences. The resultant sequences were screened for quality, and ≥70 base pairs were selected for bioinformatics analysis. All sequences were classified using the NCBI BLAST and SILVA databases. Distance calculation, operational taxonomic units cluster, rarefaction analysis, and estimator calculation (α-diversity and β-diversity) were performed by the MOTHUR program [37].

### Statistical analysis

Values presented are expressed as mean ± SEM. Statistical analyses were performed using SPSS software version 17.0 (SPSS Inc., Armonk, New York, USA). Escape path length and escape latency in MWMT were analyzed using two-way analysis of variance (ANOVA). Other data were analyzed using one-way ANOVA followed by post-hoc Tukey’s test, Student’s *t*-test, or Fisher’s exact test. *P*-values less than 0.05 were considered statistically significant.
